# Terri and *G&D*: celebrating 50 years of enlightened scientific judgment

**DOI:** 10.1101/gad.350441.123

**Published:** 2023-01-01

**Authors:** Robert Tjian

**Affiliations:** Department of Molecular and Cell Biology, Li Ka Shing Center for Biomedical and Health Sciences, University of California, Berkeley, Berkeley, California 94720, USA

## Abstract

GUEST EDITOR

It is difficult to imagine the scientific publishing landscape without Terri Grodzicker at the helm of *Genes & Development*. Here we celebrate over 50 years of Terri's many accomplishments and awesome staying power, first as a bench scientist with a stellar decades-long career in molecular genetics in Boston and Cold Spring Harbor, and then as Editor of *G&D* since 1989—where did the time go?

My high regard for Terri dates back to my very first weeks at Cold Spring Harbor Laboratory in 1976, where I, as a newly minted PhD trained in bacterial and phage biochemistry, was attempting to do mammalian tissue culture. With firm but gentle guidance from Terri, I finally managed after some weeks not to contaminate CV1 monkey cells with bacteria (we think the culprit was *Bacillus subtilis* spores hitching a ride on my clothes). Terri and I became lifelong friends, colleagues, and collaborators, and eventually comentors of graduate students after I moved to Berkeley for my first and only faculty position. In those 3 years at CSHL, I came to appreciate Terri's exacting scientific standards, her exceptional ability to consistently formulate the original ideas that guided her own work, and her capacity to perceive the significance of discoveries by others. For as long as I have known her, and in the many contexts I have seen her operate, I have been struck by the high quality of her work and character.

By the mid to late 1980s, with rapid advances unraveling various aspects of gene regulation and developmental biology enabled by the latest tools of recombinant DNA, molecular cloning, protein purification, and genome sequencing, scientific publication was entering a period of unprecedented growth. The journal *Cell* under Ben Lewin had transformed publication of molecular biology and biochemistry, and meanwhile the world of biology was being upended by the biotech revolution occurring at Genentech, Cetus, Amgen, and Chiron. This rapid acceleration in the pace of biological research, driven by both the emerging biopharma industry and the burgeoning academic research enterprise, also entailed an expansion of scientific publication in the biological sciences and an opportunity to launch new scientific journals. Jim Watson, as Director of CSHL, instantly recognized the importance of establishing high-quality journals beyond *Cell*, *Nature*, *Science*, and the *Journal of Molecular Biology*. It seemed an opportune moment to launch a new journal focused on both genotype and its relationship to phenotype—and so he did, calling it *Genes & Development*.

At first, *G&D* was run by CSHL staff member Mike Matthews, who worked on a part-time basis because he was not prepared to completely give up his own research at the bench. However, it soon became apparent to many that for *G&D* to reach its full potential and be competitive in attracting the best papers, its Editor would need to be all in. At the same time, many of us recognized that it would be ideal to recruit an experienced, accomplished, and exceptionally astute research scientist rather than a professional journal editor. We hoped to install an actively practicing scientist with an uncanny ability to recognize cutting-edge research, an impeccable taste in science, and, most importantly, an ability to discern merely solid science from field-launching transformational discoveries. Those of us who knew Terri's research accomplishments, her intellectual breadth, and her intuition for identifying great science and talent felt that she, if willing, could develop into a standard-bearing Editor that would mold *G&D* into a top science journal of its time.

Terri's position as a staff member at CSHL offered an additional and unique advantage. Although she would inevitably be pulled away from her own hands-on research to become a full-time Editor, by being an active member of the CSHL community, she could still maintain and even broaden her knowledge of cutting-edge research as a scientist–Editor. CSHL's regular, intense, world-class scientific conferences, attended by the best and brightest from across the globe, would further sharpen her acute scientific intuition and allow her to stay abreast of rapidly evolving advances in multiple emerging fields in the biological sciences. This way, she would have the same advantage that was so critical for Ben Lewin, who, during the early days of *Cell,* stayed current and met the rising stars in emerging fields by being connected to MIT, Harvard, and the wider Boston/Cambridge community. As it turns out, Terri not only took full advantage of this catered feast of top science and scientists at her disposal as Editor of *G&D* embedded in CSHL, but was actively engaged in organizing many of the scientific meetings herself, and later participated in the formation of the School of Biological Sciences for PhD students.

Well, despite these obvious advantages available to Terri, it was nonetheless a difficult decision for a productive bench scientist to shut down her lab and take on this potentially risky venture to become Editor of a new journal. It took many conversations with close colleagues before Terri made the leap to accept the position as full-time Editor of *G&D*. When that happened, many of us felt optimistic that *G&D* would become a top-tier journal positioned to make a big impact and help propel our rapidly advancing field of gene regulation and developmental biology. However, few if any of us could have then imagined how Terri would truly transform *G&D*, empower many careers, and have a significant hand in fostering numerous transformative discoveries as Editor.

I will not dwell on the many great papers Terri has published in her more than 35 years as the gatekeeper at *G&D*, since I am sure many others who are contributing to this special issue will amply enumerate them. However, I'd like to recount just a few of my own experiences interfacing with Terri as Editor that epitomize what I find is a profoundly critical and yet rapidly vanishing quality among the editorial leadership of top journals today. In addition to seeking out rising talent from early career scientists at CSHL summer meetings, what has always separated Terri from most other journal editors is her singular ability to quickly capture the key insight about new discoveries, balancing at once deep scientific knowledge, healthy skepticism, sage advisors, timely decision-making, and bold vision to publish novel and even paradigm-shifting findings and ideas. More often than not, her editorial suggestions helped improve our manuscripts and elevated the high standard that we all aspire to achieve in our publications.

Having published 46 papers in *G&D*, it was not easy to pick a few case studies, but two examples that strike close to home are the Pugh and Tjian (1991) and Tanese et al. (1991) *G&D* articles. This work reported on the incipient notion that the RNA polymerase II preinitiation complex must include some previously unrecognized components beyond sequence-specific DNA-binding activators and subunits of the core promoter recognition complex (i.e., TBP, TFIIA, TFIIB, TFIIE, TFIIF, etc.). Terri early on saw the potential impact of the idea that in addition to TBP, there were components we called TBP-associated factors (TAFs) that make up an integral part of the TFIID complex. Hence, Terri published several of our first papers on human and *Drosophila* TAFs, having recognized their potential role in both TATA-box-containing and TATA-less promoters despite a fair amount of initial skepticism about their importance (especially from the yeast transcription community, which had largely failed to detect TAFs). Indeed, it was not generally accepted at the time that TBP may not be the only or even the most functionally important subunit of TFIID. Likewise, the idea that a TAF–TBP ensemble might serve as a new class of coactivators needed to communicate between sequence-specific enhancer-bound transcription factors and the core promoter machinery seemed overly complex and was deemed somewhat “inelegant.” Terri's willingness to take the risk and publish these findings speaks volumes about her vision and boldness to buck the popular trend. Most critically, as Editor, she was knowledgeable and self-assured enough to recognize potential biases of reviewers and the conviction to override misguided recommendations—an intellectual independence that is indispensable for good editorial judgment but is sadly in short supply these days.

By way of further illustration, Terri's perceptive intuition and willingness to go against the grain can be seen in two review papers published more recently by *G&D* from our lab. One review challenged the invocation of phase separation in high-profile journals engorged with reports of the often misinterpreted and trendy role of liquid–liquid phase separation (LLPS) in cellular processes. Indeed, basic experiments to rigorously demonstrate even the occurrence of bona fide LLPS in vivo had been largely absent from the majority of such publications. Equally glaring was the propensity to infer mechanism or causation from the mere presence of puncta in cells without ascertaining what function phase separation performs under physiologically relevant cellular contexts for diverse biological reactions, including transcription. The McSwiggen et al. (2019) *G&D* review laid bare many of these issues and triggered some in the field to reflect more rigorously about the true role of LLPS and even to question whether some observed puncta are, in fact, phase-separated compartments at all.

Another example of Terri's bold editorial thinking was her willingness to publish a rather unconventional model that challenges the long-held and seemingly established concept of transcription factor-mediated DNA looping as the primary mechanism for long-distance enhancer–promoter interactions. The TF activity gradient (TAG) model proposed by Karr et al. (2022) offers a novel solution to the two key problems of enhancer–promoter communication: What signal is transmitted from distal enhancers to promoters, and by what mechanism does the transmission occur? Karr et al. (2022) propose that the transmitted signal could be TFs that are post-translationally modified by cofactors such as p300, and the activated TFs then diffuse to nearby promoters, where they act via an enzymatically time-limited diffusion mechanism originating from the distal enhancer point source. Thus, direct contact between enhancer-bound factors and promoter elements may be dispensable. Here again, Terri as Editor of *G&D* had to exercise some courage to risk publishing innovative ideas that squarely went against the grain of popularly accepted paradigms.

In the early days when CSHL was seeking an inaugural editor for *G&D*, we debated who might be a candidate with the “decisiveness and backbone” to go against engrained trends and make tough, sometimes risky decisions. Having collaborated closely with Terri on a variety of successful research projects, I was convinced she had that strength of conviction as well as the scientific chops to transcend the conventional and strike into new directions as Editor of *G&D*. After several decades with Terri at the helm, we all can see that she not only has exercised her intuition for good science, but most importantly, has time and again acted on her strength of conviction. For this, the many students, postdocs, and early career scientists seeking to publish paradigm-shifting research and bleeding-edge findings thank her. We all owe Terri a debt of gratitude for helping to launch many careers rather than crushing aspirations to toe the line of popularity and trendy publishing.

The current landscape of traditional scientific publishing is in clear turmoil; some would say it is a badly broken system exacerbated by the increasing challenge of finding truly savvy, experimentally sophisticated Editors with astute scientific judgment among the ever-expanding ranks of commercially motivated scientific journals. Once again, we find ourselves at a critical juncture of the scientific enterprise as top-tier journals pose major obstacles to rapidly and robustly vetted papers that are not merely chasing the latest trends. We can only hope that a few next-generation Terri Grodzickers will step up to steer scientific publications into a more effective, transparent, and equitable future. These are undoubtedly big shoes to fill, and we will profoundly miss the reliable, inspired judgment and good taste of Terri Grodzicker.
